# Laboratory observations of slow earthquakes and the spectrum of tectonic fault slip modes

**DOI:** 10.1038/ncomms11104

**Published:** 2016-03-31

**Authors:** J. R. Leeman, D. M. Saffer, M. M. Scuderi, C. Marone

**Affiliations:** 1 Department of Geosciences, The Pennsylvania State University, 522 Deike Building, University Park, Pennsylvania 16802, USA; 2 Dipartimento di Scienze della Terra, Sapienza Università di Roma, Piazzale Aldo Moro 5, 00185 Rome Italy

## Abstract

Slow earthquakes represent an important conundrum in earthquake physics. While regular earthquakes are catastrophic events with rupture velocities governed by elastic wave speed, the processes that underlie slow fault slip phenomena, including recent discoveries of tremor, slow-slip and low-frequency earthquakes, are less understood. Theoretical models and sparse laboratory observations have provided insights, but the physics of slow fault rupture remain enigmatic. Here we report on laboratory observations that illuminate the mechanics of slow-slip phenomena. We show that a spectrum of slow-slip behaviours arises near the threshold between stable and unstable failure, and is governed by frictional dynamics via the interplay of fault frictional properties, effective normal stress and the elastic stiffness of the surrounding material. This generalizable frictional mechanism may act in concert with other hypothesized processes that damp dynamic ruptures, and is consistent with the broad range of geologic environments where slow earthquakes are observed.

## Abstract (web summary)

Slow earthquakes, where fault slip is slow, can be large and may help trigger regular earthquakes, but the mechanics of slow slip are not fully understood. Leeman *et al.* show through laboratory experiments that slow slip behaviour on faults is controlled by the frictional dynamics of the surrounding material.

## Graphical abstract

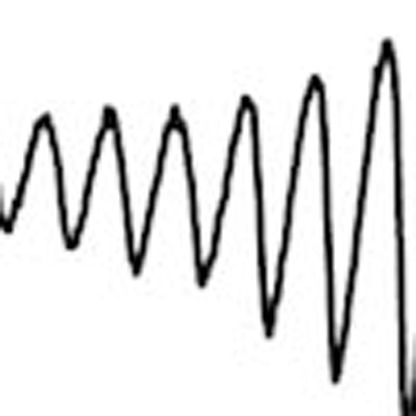

Slow earthquakes are a mode of self-sustained fault rupture in which slip accelerates but does not reach rates sufficient to radiate high-frequency seismic energy^1^,^2^. Seismic and geodetic observations reveal that slow-slip and the related phenomena of low-frequency earthquakes and non-volcanic tremor define a spectrum of slip behaviours that unfold over timescales ranging from seconds to months^2^,^3^,^4^,^5^,^6^. Slow earthquakes can be large, in some cases equivalent to M7+ earthquakes, and they may play a role in stress transfer and thus triggering of damaging regular earthquakes^7^. Slow earthquakes have also been observed as precursors to regular earthquakes and thus they may provide insight into the processes of earthquake nucleation^8^,^9^. Although geophysical observations have resolved fine details of slow earthquake slip and propagation rates of tectonic fault tremor^6^,^8^,^9^,^10^, the fundamental and controlling mechanics of these phenomena remain enigmatic.

Regular earthquakes have long been understood in terms of stick–slip failure dictated by frictional and elastic properties of the Earth's crust^11^. Laboratory studies have provided key insights into the physics of fault failure and its dynamics, both for repeating earthquake-like stick–slip failure and for more complex slip behaviours. For example, previous works have reported a range of observations including transient slip, oscillatory sliding behaviour and dynamic rupture at sub-Raleigh and supershear propagation speeds^12^,^13^,^14^,^15^,^16^,^17^,^18^. Transient and oscillatory behaviour have been interpreted as analogues for premonitory slip prior to earthquakes or transient aseismic slip^12^,^18^,^19^.

Despite their relevance to natural fault zones and slow earthquakes, detailed laboratory observations of repetitive slow-slip transients are few and do not include systematic studies. These behaviours have been reported in some experimental work^12^,^14^,^15^, but have been interpreted and modelled in the context of specific fault rheologies, using so-called ‘designer' friction laws. In one form of these laws, slow stick–slip is produced by an increase in frictional resistance with slip velocity, such that instability is quenched during acceleration^14^,^15^,^19^. Other explanations for slow earthquakes have focused on processes that may arrest slip acceleration during earthquake nucleation, including dilatancy hardening^20^,^21^, transitional frictional behaviour as a function of slip^22^ or slip rate, and fault zone heterogeneity. Some numerical simulations successfully predict complex slip behaviour, including oscillatory behaviour and the emergence of periodic slow slip^20^,^23^. Two-dimensional (2D) numerical models also show promise in reproducing natural events, with fewer free parameters than multiple state variable models^24^.

To date, the origin of slow earthquakes has been explored largely via seismic or geodetic data or through numerical experiments with only sparse, isolated laboratory observations to probe the underlying mechanics. Although theoretical models can explain the emergence of slow-slip transients under certain conditions or for specific frictional rheologies^20^,^21^,^23^, a fundamental mechanical explanation for these events remains elusive. Yet, slow modes of fault rupture are observed in a variety of tectonic and geologic settings, and with a wide range of durations, raising the question as to whether they arise from a universal mechanism^6^,^25^.

Although many fault zones are rich in phyllosilicate minerals, which have been shown to exhibit both rate-weakening and rate-strengthening behaviour under conditions comparable to those expected *in situ* in the seismogenic crust^24^,^26^,^27^, we focus on quartz gouge to investigate the systematics of frictional failure, because it is a well-studied material that is common in natural faults, and is thought to play a key role in controlling their slip behaviour^27^,^28^. Quartz gouge also exhibits frictional properties that enable us to probe the stability boundary using geophysically relevant values of normal stress and sliding rates. This allows a detailed investigation of the frictional dynamics of slow slip, which provides a robust and generalized framework to apply to tectonic fault zones.

Here we describe laboratory experiments that reproduce the full spectrum of fault slip behaviours under geophysically relevant conditions of normal stress and fault composition, and which illuminate their underlying physics. Our experiments are designed to explore the full range of slip stability, as described by the stability parameter *κ=k/k*_c_, from *κ>1* (inherently stable slip) to dynamic stick–slip (*κ*<<*1*). Consistent with previous works^14^,^18^,^23^ near the stability boundary, *κ≈1*, we observe complex slip patterns that precede slow slip. We document a systematic and robust relationship between departure from the stability threshold, slip velocity and duration of repetitive failure events. Our experimental results, to the best of our knowledge, are the first complete and systematic study to investigate the full spectrum of slip behaviours from slow to fast events, as observed for tectonic faults.

## Results

### Mechanical behaviour

**Figure 1:**
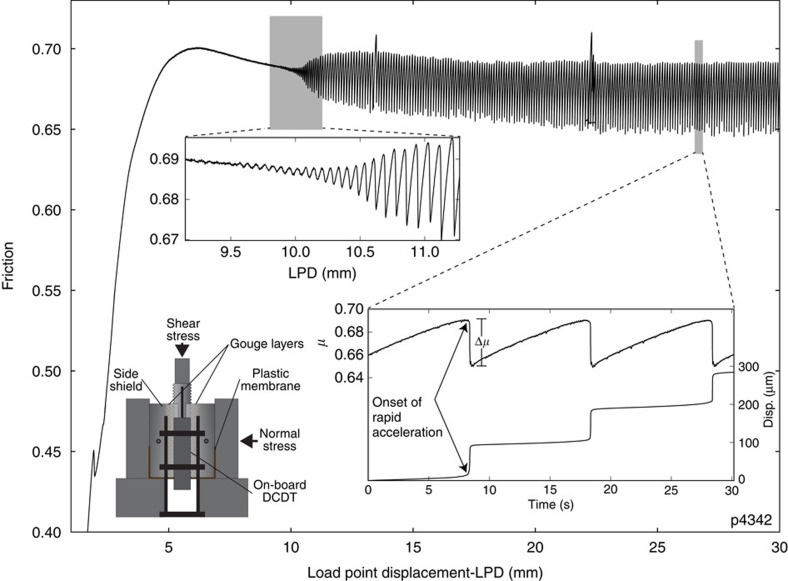
Experimental run plot. Friction data for one experiment (p4342) at a normal stress of 12 MPa and shearing rate of 10 μm s^−1^. The upper inset shows spontaneous emergence of unstable slow slip. Stick–slip amplitude increases gradually over a few millimetres before reaching steady state. The lower right inset shows details of fault slip events, note the gradual acceleration at the start of each failure event. The lower left inset shows the double direct shear configuration and locations of displacement transducers. Spikes at 13 and 22 mm displacement are due to brief pauses in shearing to reset displacement transducers.

**Figure 2:**
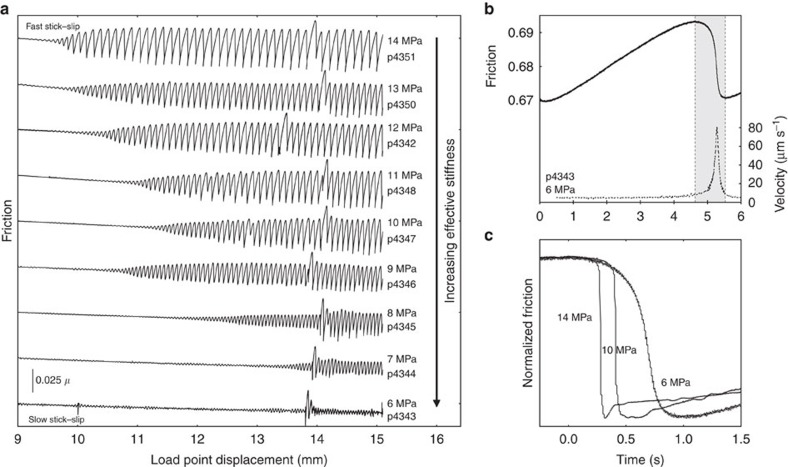
Spectrum of fault slip behaviour. (**a**) Friction data for experiments (p43XX run numbers) at different effective shear-loading stiffness *k′*=*k/σ*_n_*′*. Friction data are offset vertically for clarity. The emergence of slow stick–slip occurs at lower shear displacement, and stick–slip amplitude increases, for higher normal stress experiments. The spikes in friction at 13–15 mm are due to frictional aging caused by brief pauses in shearing to reset displacement transducers. (**b**) Details of friction (solid line) and velocity (dashed) during a stick–slip event with a peak slip velocity of ≈80 μm s^−1^, only a few times that of the background loading velocity of 10 μm s^−1^. (**c**) Stick–slip events have systematically longer duration at lower normal stresses. Slip accelerates more slowly and event durations are correspondingly longer than at higher normal stress.

In our experiments, gouge layers initially exhibited stable sliding, followed by the emergence of repeating slow stick–slip events (Figs 1 and 2a). The slow-slip events arose gradually, over an interval of up to 1.5 mm, and then increased in amplitude over as few as 10–20 slip events before reaching a mechanical steady state, characterized by relatively uniform recurrence intervals and friction drops, up to the maximum imposed displacements of ≥50 mm. For our layers, which were 3-mm-thick prior to shear, this corresponds to shear strains of 30–50. Each slow-slip event began with a gradual acceleration and culminated in a slip event and stress drop (Fig. 1).

### Stick–slip events

Our experimental results are consistent with theory, numerical experimentation^20^,^23^ and with existing lab data for stick–slip^11^. We document a spectrum of stick–slip behaviours in experiments conducted over a range of normal stresses (Fig. 2a). At low normal stress (6 MPa) and close to the stability transition described by equation (1), slip events have systematically longer duration and smaller stress drops than their higher normal stress counterparts (Fig. 2c). Details of the friction records for slow events show that slip begins gradually, well before the peak strength is reached and then accelerates during the stress drop (Fig. 2b). The maximum slip velocities for slow-slip events are in the range of 50–100 μm s^−1^, and slip speed increases systematically with increasing normal stresses, which leads to increasingly unstable behaviour (equation (1)). For the lowest values of normal stress that produced repeating transient slip events, we measured peak slip velocities of only a few 10's of μm s^−1^, on the order of the driving velocity. For a normal stress of 14 MPa, we observed audible fast stick–slip events with slip velocities >2 mm s^−1^.

## Discussion

The short duration, audible high slip velocity events are manifestations of dynamic instability and represent laboratory analogues of regular, fast earthquakes^11^. Likewise, we posit that the observed spectrum of slow to fast stick–slip events in our experiments are representative of the spectrum of slip behaviours observed on tectonic faults, including repeating slow-slip events and low-frequency earthquakes^4^,^6^. Near the stability transition, we also document complex and chaotic behaviours including period doubling and transient variations in stick–slip amplitude with long-period modulation (Fig. 2a), consistent with theoretical predictions 23.

**Figure 3:**
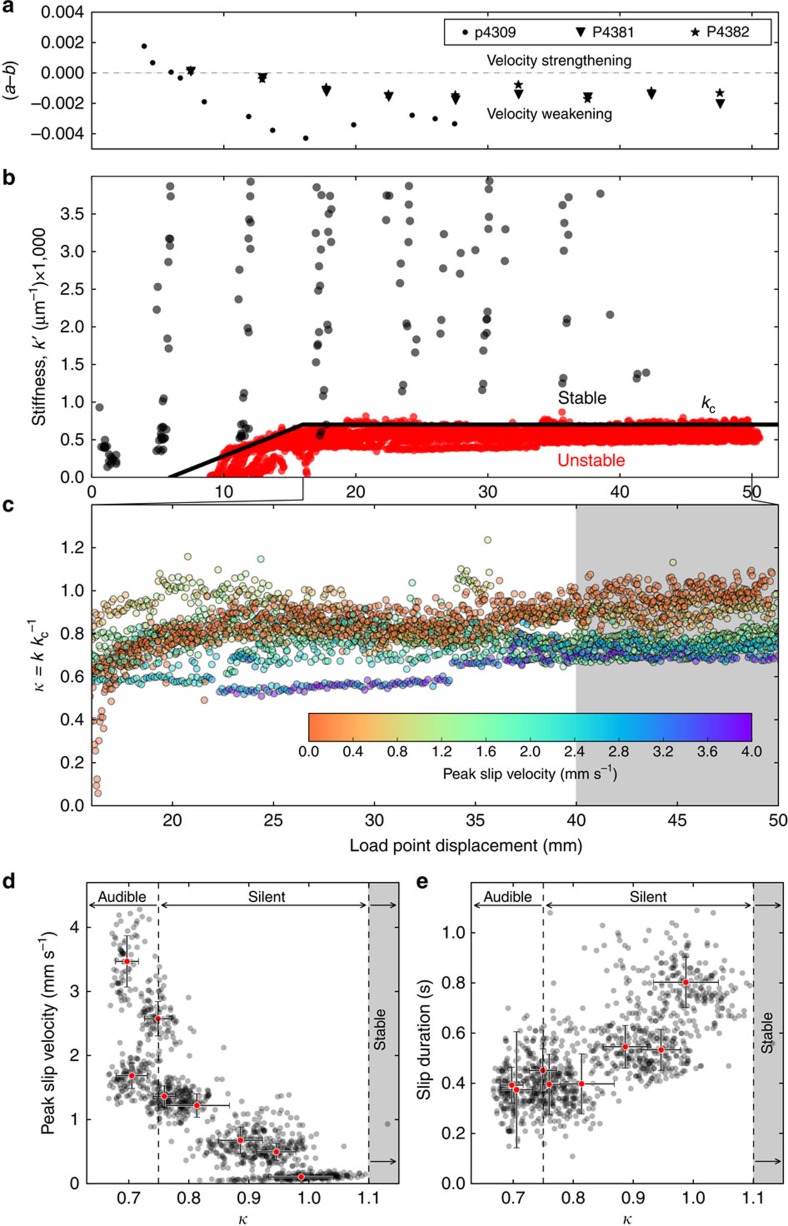
Stick–slip event properties. (**a**) The friction rate parameter *(b−a)* transitions from velocity strengthening to velocity weakening at ∼5–7 mm displacement. (**b**) Data from 29 experiments showing effective friction stiffness *k′*= *k/σ*_n_*′* as a function of shear displacement for stable sliding (black dots) and stick–slip events (red dots). The heavy black line defines the evolution of *k*_c_*′* based on the distinction between stable sliding and stick–slip. (**c**) Data for unstable slip events shown in **b** are colour coded by peak slip velocity and shown as a function of shear displacement. Stick–slip is slowest for *κ*∼1. The 40–50 mm interval marked by the grey box denotes data used to compile stick–slip properties. (**d**) Stick–slip event velocity and (**e**) duration as a function of normalized critical stiffness *κ* =*k/k*_*c*_. Black dots show data from events in the displacement interval 40–50 mm for eight experiments; red dots show mean values ±1 s.d. for each experiment.

To investigate the mechanics of slow stick–slip events, we carefully measured both the elastic loading stiffness *k* and the critical stiffness *k*_c_ in each of our experiments. We measured *k* directly from the loading curves of stick–slip events and from unload/reload cycles (Supplementary Fig. 1). Stiffness increases with shear displacement up to 15 mm, and then reaches an approximately constant value (Fig. 3; Supplementary Fig. 1). The increase in stiffness with shearing is consistent with shear-enhanced compaction and granular comminution during the first few millimetres of slip^29^. As noted above, we measure *k*_c_ directly from the parameters in Equation (1) using velocity step experiments (Fig. 3a, Supplementary Fig. 2), and also empirically using the value of *k′* at the observed transition between unstable and stable slip (black line, Fig. 3b). The empirically defined threshold stiffness increases with displacement and reaches a steady value of ≈7 × 10^−4^ μm^−1^ at a displacement of ∼16 mm, equivalent to a shear strain of ∼5–6 (Fig. 3b). Direct measurements of *k*_c_ yield similar values (6–7 × 10^−4^ μm^−1^; Supplementary Fig. 2), and also show that *k*_c_ increases dramatically within the first ∼10 mm of shear displacement. This is due to the combined effects of increasingly velocity-weakening friction (Fig. 3a) and decreasing critical slip distance *D*_c_ with shear strain (Supplementary Fig. 2). The evolution of *(b−a)* is consistent with inferred shear localization and with the observation that unstable slip emerges after a finite shear strain (Fig. 1). The shear displacement needed for the emergence of slow slip decreases with increasing *σ*_*n*_*′* (Fig. 2a), consistent with enhancement of shear localization and fabric development at higher *σ*_*n*_*′*.

Taken together, our direct (Fig. 3a, Supplementary Fig. 2) and independent (Fig. 3b) measurements of *k*_c_*′* and *k′* (Supplementary Figs 1 and 2) show that stick–slip event velocity and duration vary systematically as a function of distance from the stability threshold. The slowest events occur for *κ≈1*, with progressively faster events for lower values of *κ* (Fig. 3d,e). The peak slip velocity and stick–slip duration for all events, measured after reaching a steady state (Fig. 3c, shaded area), define a complete spectrum of slip behaviours between stable sliding and fast stick–slip (Fig. 3d,e). For *κ<0.7*, slip velocities of several mm s^−1^ were associated with audible failure events (Fig. 3d). For values of *κ* approaching 1, the duration of slow-slip is in the order of seconds (not producing any audible emissions in the range of human hearing), with lower peak slip velocities (Fig. 3d,e). The amplitude of the stick–slip events is systematically lower for the slow events (Fig. 2), consistent with seismic and geodetic observations for tectonic faults^4^,^6^,^30^.

Our data show that the full spectrum of stick–slip behaviours can occur over a relatively narrow range of conditions near the stability phase boundary, and further that the mode—and slip velocity—of unstable sliding vary predictably as a function of departure from this threshold. Although the 1D spring-slider model is simplified relative to the geometry and rheology of natural fault systems, the predicted stability regimes are remarkably consistent with our laboratory experimental data. It is also consistent with theoretical models that incorporate more complex 2D fault geometries and elastic interactions^20^, suggesting that to first order, the mechanics and dynamics of these systems are captured by this relatively simple and elegant model^15^,^18^,^23^,^29^,^31^.

In total, our results illuminate the key ingredients required for slow earthquakes. Relative to areas where regular earthquakes occur, *k*_c_ must remain sufficiently small that it does not greatly exceed the local fault stiffness *k*. This can occur for specific frictional properties—small *(b−a)* or large *D*_c_—as may be the case at the upper and lower edges of the seismogenic zone or in areas of complicated fault zone architecture^20^. This condition would also be favoured by low effective normal stress, as has been suggested in a wide range of settings^8^,^9^,^31^,^32^,^33^,^34^. In addition, we suggest that the mode of fault slip should evolve as tectonic faults accumulate shear strain, or through the earthquake cycle, due to progressive changes in fault stiffness and frictional constitutive properties^32^,^34^. Finally, because fault stiffness is proportional to the ratio of shear modulus to rupture nucleation patch size, we expect that regions of large, coherent creep slip, which effectively reduce *k*, would favour nucleation of slow earthquakes.

Our results support previous hypotheses about the role of transitional frictional behaviour in driving complex fault slip behaviours^20^,^23^,^31^,^32^,^33^. It is likely that transitional frictional behaviour may act in concert with additional processes acting locally within a fault zone to produce the observed spectrum of slip behaviours. A wide range of key natural factors, such as compliant and evolving damage zones, low effective normal stress associated with elevated pore fluid pressure and fault evolution are all captured by the stability parameter *κ=k/k*_c_. Ultimately, our results suggest that slow earthquakes and transient fault slip behaviours arise from the same governing frictional dynamics as normal earthquakes, and provide a unified view of the spectrum of tectonic fault slip behaviours.

## Methods

### Experimental apparatus

Experiments were performed in a servo-controlled biaxial shearing apparatus using the double direct shear configuration (Fig. 1). Displacements on the normal and shearing axes were measured by Direct Current Displacement Transducers (DCDTs), referenced at the load frame and ram nose. The displacement of the shearing block was measured with DCDTs referenced at the end-platen and the top and bottom of the shearing block (Fig. 1). Loads applied to the sample were measured with strain gauge load cells. All transducers are calibrated with instruments and methods traceable to NIST.

### Sample preparation

Samples were prepared using steel or titanium side blocks and steel or acrylic central shearing blocks (Supplementary Table 1). The forcing blocks were grooved 0.8 mm deep at 1 mm spacing to eliminate shear at the boundary. We used Min-U-Sil 40 powdered silica (US Silica Co.) to simulate granular fault gouge. The product is 99.5% SiO_2_, with traces of metal oxides, and has a median grain diameter of 10.5 μm. Samples were constructed as 3-mm-thick layers, and with 10 × 10 cm frictional contact area. Layers were prepared and sheared under 100% relative humidity at room temperature.

### Testing procedure

After samples were placed in the testing machine, a constant normal stress was applied and maintained constant using force-feedback servo control. Samples were allowed to compact and accommodate grain rearrangement before shearing began. Shear was induced by imposing a displacement rate on the central forcing block (Fig. 1), using a feedback servo control. The displacement rate was maintained constant at 10 μm s^−1^ for the majority of our experiments (Supplementary Table 1), and velocity step tests were used to determine the friction rate parameters *(a−b)* and *D*_c_.

We used a range of shear-loading stiffnesses *k* given by the summation, in series, of the apparatus stiffness, the stiffness of the loading blocks and the stiffness of the layers of fault gouge. The effective loading stiffness of the testing machine *k′*=*k/σ*_n_*′* was altered by using a compliant central forcing block and by changing the applied normal stresses (Fig. 2a). We measured *k* in experiments using a least-squares linear fit to friction versus shear displacement for the interval *μ*=0.3−0.4 and from the elastic loading portion of stick–slip events (Supplementary Fig. 1). Rate-and-state friction parameters were determined (Supplementary Fig. 2) using an iterative singular value decomposition technique.

### Frictional stability

In the context of frictional stability, the criterion for unstable stick–slip in a simplified 1D system is defined by the interaction between loading system stiffness *k* and a rheologic critical stiffness of the fault, *k*_c_:


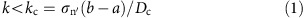


where *(b−a)* is the friction rate parameter and *D*_c_ is the critical slip distance^29^. Negative rate parameters, *(b−a)<0*, indicate velocity-strengthening behaviour, which is inherently stable. Positive values of *(b−a)* indicate velocity-weakening friction and are a prerequisite for instability and earthquake nucleation. Within the velocity-weakening regime, if the condition in equation (1) is satisfied (that is, stiffness of the loading system, *k*, is less than the critical stiffness; *k*<*k*_c_), instability occurs because the fault weakening rate, *k*_c_, exceeds the rate of elastic unloading, leading to a force imbalance. For stiffer systems (that is, *k*>*k*_c_), in which elastic unloading outpaces frictional weakening, sliding is stable. For convenience, we normalize the stiffness and critical stiffness by the normal stress, appending a prime symbol to denote this; *k′=k/σ*_n_*′* and *k*_c_*′=k*_c_*/σ*_n_*′*

We selected values of *k* and normal stress for our experiments to span the stability boundary for our fault gouge. To achieve this, we made careful measurements of the evolution of *k* and *k*_c_ with shear strain (Supplementary Fig. 1). For a given set of frictional properties, defined by *(b−a)* and *D*_c_, the ratio *k/σ*_n_*′* defines an effective system stiffness, *k′* (μm^−1^), that governs sliding stability. In our experiments, the testing machine, sample assembly and gouge layer together determine the system stiffness. We varied *k* using different forcing block materials (Supplementary Table 1) and *k′* via the normal stress.

## Additional information

**How to cite this article:** Leeman, J. R. *et al.* Laboratory observations of slow earthquakes and the spectrum of tectonic fault slip modes. *Nat. Commun.* 7:11104 doi: 10.1038/ncomms11104 (2016).

## Supplementary Material

Supplementary Information Supplementary Figures 1-2 and Supplementary Table 1.
