# Supplementary material for: Laboratory observations of slow earthquakes and the spectrum of tectonic fault slip modes
Source: Nat Commun. 2016 Mar 31;7:11104. doi: 10.1038/ncomms11104 (PMC4821871; doi:10.1038/ncomms11104)
Supplement: Supplementary Information — Supplementary Figures 1-2 and Supplementary Table 1. [file ncomms11104-s1.pdf]

Supplementary Figures

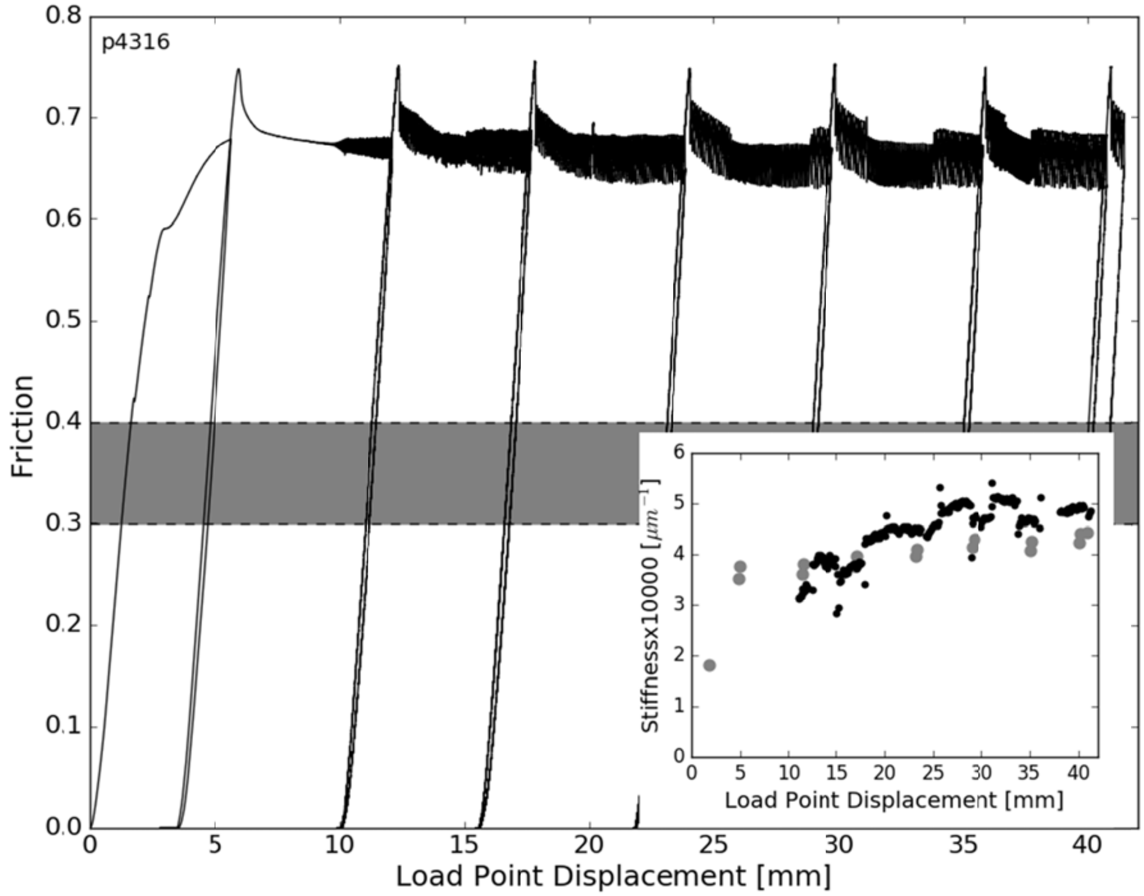

Supplementary Figure 1 – Stiffness during an experiment. Friction data for one complete experiment (run p4316) with shear load-unload cycles. Slow slip events emerge spontaneously at a displacement of ~8.5 mm. (Inset) Stiffness measured from unload-reload cycles in the range μ=0.3-0.4 (large grey circles) and from the slope of the elastic loading segments of individual stick-slip events (small black circles). Both measurements indicate an initial increase in stiffness, caused by shear enhanced compaction. In this experiment we explored multiple shearing rates during slow slip (e.g., for events in the range 27-29 mm and 31-33 mm), which had a clear effect on stick-slip amplitude and stiffness.

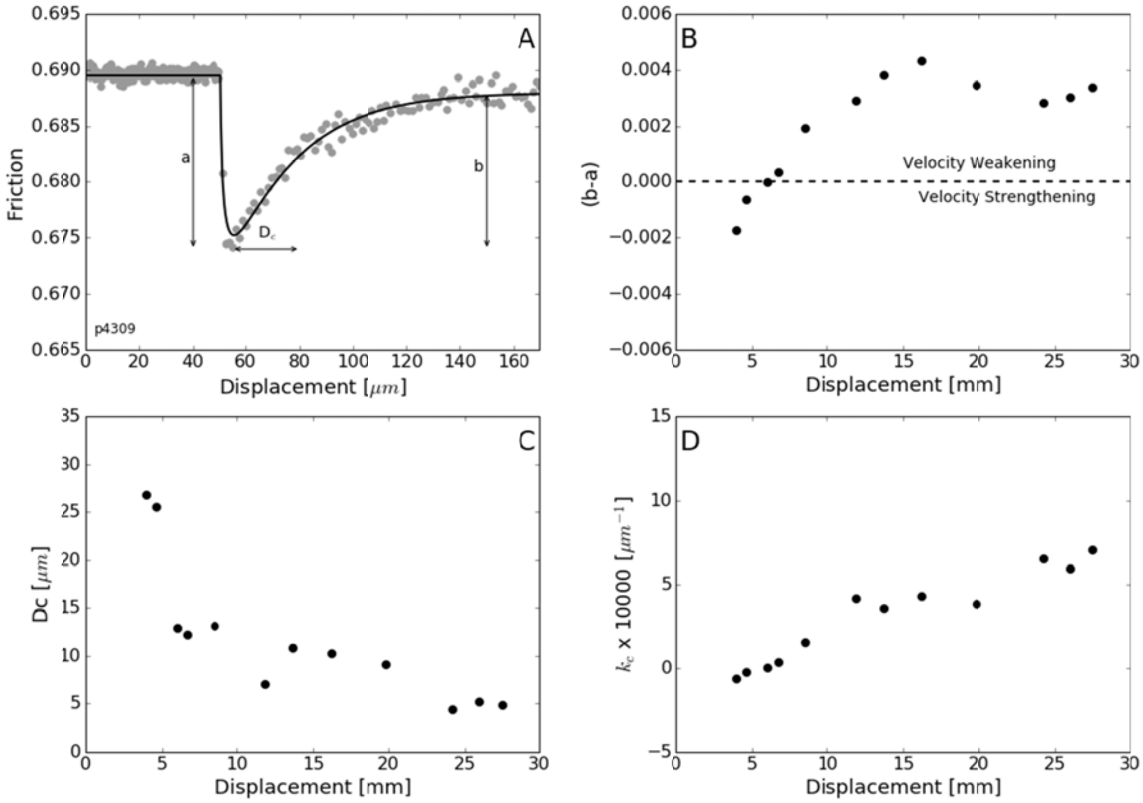

Supplementary Figure 2 – Rate and State Parameters. Data and modeling procedure for obtaining rate-and-state friction parameters and their variation with shear displacement. A) Data from experiment p4309 (grey data points) and model (solid line) for a velocity step test from 10 to 1 µm s^{-1}, with illustration of the friction direct effect *a*, evolution effect *b*, and critical slip distance D_c. B) Evolution of (*b*-*a*) with shear displacement (see also Figure 3A). Note the transition from rate strengthening to rate weakening at ~8 mm displacement, after which a steady-state behavior is reached. C) The critical slip distance decreases with shear displacement as frictional steady-state is reached. D) Evolution of the critical stiffness (k_c) with slip.

Supplementary Tables

Experiment	Blocks	Normal Stress [MPa]	Humidity [%]	Comments	Unload/Reload Cycles
p4224	Ti/Acrylic	5	16	Stable – Vel. Steps	N
p4228	Steel	4	100	Stable – Slide Hold	N
p4229	Ti/Acrylic	4	100	Failed	N
p4248	Ti/Acrylic	4	100	Stable – Vel. Steps	N
p4249	Ti/Acrylic	4	100	Stable – Vel. Steps	N
p4267	Ti/Acrylic	2	100	Stable – Vel. Steps	Y
p4268	Ti/Acrylic	8	100	Slow-Slip	Y
p4269	Steel	4	100	Stable – Vel. Steps	Y
p4270	Steel	2	100	Stable – Vel. Steps	Y
p4271	Ti/Acrylic	2	100	Stable – Vel. Steps	Y
p4272	Ti/Acrylic	8	100	Slow-Slip	Y
p4273	Steel	8	100	Stable – Vel. Steps	Y
p4309	Steel	8	100	Stable – Vel. Steps	Y
p4310	Ti/Acrylic	8	100	Slow-Slip	Y
p4311	Ti/Acrylic	8	100	Slow-Slip	Y
p4312	Steel/Acrylic	8	100	Slow-Slip	Y
p4313	Ti/Acrylic	8	100	Slow-Slip	Y
p4314	Steel	12	100	Stable – Vel. Steps	Y
p4316	Ti/Acrylic	12	100	Stick-Slip	Y
p4317	Steel/Acrylic	12	100	Stick-Slip	Y
p4327	Steel	6	100	Stable – Vel. Steps	Y
p4328	Ti/Acrylic	6	100	Slow-Slip	Y
p4329	Ti/Acrylic	6	100	Slow-Slip	Y
p4330	Steel	6	100	Stable – Vel. Steps	Y
p4338	Ti/Acrylic	4	100	Stable – Vel. Steps	Y
p4339	Steel	4	100	Stable – Vel. Steps	Y
p4340	Ti/Acrylic	8	100	Slow-Slip	Y
p4341	Steel	12	100	Stable – Vel. Steps	Y
p4342	Ti/Acrylic	12	100	Slow/Fast Slip	N
p4343	Steel/Acrylic	6	100	Slow-Slip	N
p4344	Ti/Acrylic	7	100	Slow-Slip	N
p4345	Steel/Acrylic	8	100	Slow-Slip	N
p4346	Ti/Acrylic	9	100	Slow-Slip	N
p4347	Steel/Acrylic	10	100	Slow/Fast Slip	N
p4348	Ti/Acrylic	11	100	Slow/Fast Slip	N
p4350	Steel/Acrylic	13	100	Slow/Fast Slip	N
p4351	Ti/Acrylic	14	100	Slow/Fast Slip	N
p4381	Steel	4	100	Stable – Vel. Steps	N
p4382	Ti	4	100	Stable – Vel. Steps	N

Supplementary Table 1 – Summary of experiments. Tests were conducted at 100% relative humidity to obtain repeatable results. Shear loading stiffness was varied by using different combinations of forcing blocks (steel, titanium, and acrylic) and normal stress. Unload-reload cycles were used to measure the evolution of stiffness with shear displacement.
